# 1,2,4-Triazine Sulfonamides: Synthesis by Sulfenamide Intermediates, In Vitro Anticancer Screening, Structural Characterization, and Molecular Docking Study

**DOI:** 10.3390/molecules25102324

**Published:** 2020-05-16

**Authors:** Danuta Branowska, Zbigniew Karczmarzyk, Ewa Wolińska, Waldemar Wysocki, Maja Morawiak, Zofia Urbańczyk-Lipkowska, Anna Bielawska, Krzysztof Bielawski

**Affiliations:** 1 Faculty of Exact and Natural Sciences, Siedlce University of Natural Sciences and Humanities, 3 Maja 54, 08-110 Siedlce, Poland; danuta.branowska@uph.edu.pl (D.B.); ewa.wolinska@uph.edu.pl (E.W.); waldemar.wysocki@uph.edu.pl (W.W.); 2 Institute of Organic Chemistry, Polish Academy of Sciences, Kasprzaka 44/52, 01-224 Warsaw, Poland; maja.morawiak@icho.edu.pl (M.M.); zofia.lipkowska@icho.edu.pl (Z.U.-L.); 3 Medical University of Bialystok, Department of Medicinal Chemistry and Drug Technology, J. Kilinskiego 1, 15-089 Bialystok, Poland; aniabiel@umb.edu.pl (A.B.); kbiel@umb.edu.pl (K.B.)

**Keywords:** anticancer activity, conformational analysis, molecular docking, sulfonamides, X-ray analysis

## Abstract

In this study, we synthesized novel sulfonamides with a 1,2,4-triazine moiety according to pharmacophore requirements for biological activity. All the synthesized compounds were tested in vitro to verify whether they exhibited anticancer activity against the human breast cancer cell lines MCF-7 and MDA-MB-231. Among them, two most active ones, having IC_50_ values of 50 and 42 µM, respectively, were found to show higher anticancer activity than chlorambucil used as the reference in the in vitro tests. In addition, two other compounds, which had IC_50_ values of 78 and 91 µM, respectively, exhibited a similar level of activity as chlorambucil. X-ray analysis carried out for two of the compounds confirmed their synthesis pathway as well as their assumed molecular structures. Furthermore, a conformational analysis was performed, and electronic parameters of molecules were characterized using theoretical calculations at AM1 and DFT level. Moreover, molecular docking revealed the mode of binding of the investigated 1,2,4-triazine sulfonamides with the human estrogen receptor alpha (ER*α*).

## 1. Introduction

**Figure 1:**
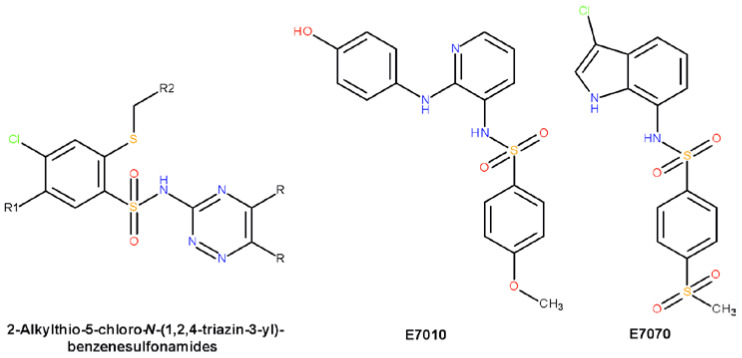
Structures of novel 1,2,4-triazine sulfonamides and sulfonamide derivatives with anticancer activity.

1,2,4-Triazines are an important class of compounds containing three nitrogen atoms. These are found to exhibit biological activity [1,2] and act similarly to biological compounds such as nucleotides or nucleosides [3]. Therefore, researchers are constantly exploring 1,2,4-triazine derivatives for novel applications, one of which involves their use as anticonvulsants for the treatment of epilepsy [4]. Currently, their focus is on analyzing the effectiveness of these compounds in treating or preventing civilization diseases such as a cancer or diabetes [5]. Furthermore, compounds with 1,2,4-triazine ring are identified as playing an important role in many different biological functions, such as inhibition of kinases, agonism/antagonism in the central nervous system (CNS), and aggregation of blood platelets [6]. However, triazines containing sulfonamide groups have not been widely studied so far. Their number is also limited, due to difficulties in their preparation. Nevertheless, there are several known examples of 1,2,4-triazine sulfonamides having anticancer activity, e.g., 2-alkylthio-5-chloro-*N*-(1,2,4-triazin-3-yl)benzenesulfonamides (Figure 1), which were able to inhibit the cancer cell growth with IC_50_ values in the micromolar or submicromolar range [7,8]. On the other hand, sulfonamides are known to display a broad spectrum of biological activities [9] and are also used as chemotherapeutic agents. Recently, a large number of new sulfonamide derivatives were investigated for anticancer activity [10,11,12,13]. These include E7010 [14] and E7070 (indisulam) (Figure 1) [15], which showed significant activity and are currently at phase I and II advanced clinical trials, respectively.

The most commonly used method to synthesize sulfonamide involves the reaction of sulfonyl chlorides with ammonia and amines in an alkaline environment [16,17]. This kind of synthesis has also been studied scientifically by our group, but the low yields prompted us to use the sulfenamide derivatives that are widely known for their medical applications [18,19,20].

Recently, we reported our achievements in the preparation of new sulfide and disulfide 1,2,4-triazine derivatives, which exhibited anticancer activity in in vitro tests [20]. The results obtained for sulfur derivatives were satisfactory, which encouraged us to design new compounds containing sulfonamide groups. With this objective, we conducted an experiment to synthesize 1,2,4-triazine sulfonamides as these compounds exhibit biological activities that are represented by many well-known drugs. 

In this paper, the synthesis and in vitro anticancer screening of new 1,2,4-triazine sulfonamide derivatives are described. X-ray analysis and theoretical calculations at AM1 and DFT level were carried out to characterize the structural and electronic parameters and conformational preferences of investigated sulfur derivatives of 1,2,4-triazine. Molecular docking studies were also performed, with the aim of linking the biological activity of the tested derivatives with their physicochemical properties by analyzing the interactions of ligands with the active site of the human estrogen receptor alpha (ER*α*). 

## 2. Results and Discussion

### 2.1. Synthesis

**Scheme 1:**
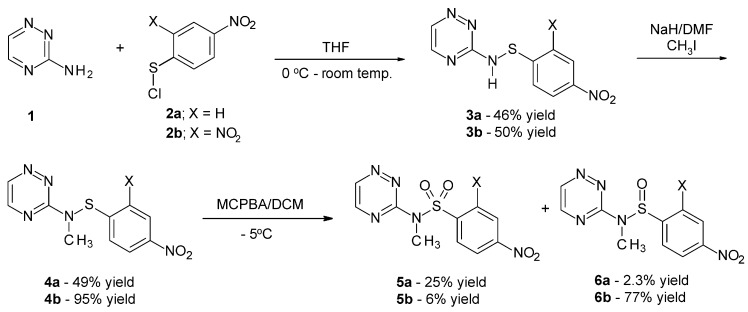
Synthesis of sulfonamides **5** and **6** starting from sulfenyl chloride **2a**,**b**.

The synthesis of 1,2,4-triazine sulfonamide derivatives was started from 3-amino-1,2,4-triazine **1.** The synthesis involved a condensation reaction between acetamidrazone with glyoxal [21]. First, amino-1,2,4-triazine was reacted with *p*-nitrobenzene sulfenyl chloride (**2a**), which led to the formation of sulfenamide **3a** in a yield of 46%. Oxidation of this compound with MCPBA did not produce sulfonamide derivative **5a**. Therefore, the secondary amine group in **3a** was alkylated to tertiary amine **4a** in a yield of 49%. Treatment of the latter with MCPBA in dichloromethane gave rise to the appropriate sulfonamide **5a** and sulfinamide **6a** in a yield of 25% and 2.3%, respectively. An analogous route was also attempted using 2,4-dinitrobenzene sulfenyl chloride (**2b**) in order to obtain *N*-sulfenylamide **3b**, which resulted in a 50% yield. In this case, the alkylation process allowed obtaining the expected compound **4b**, almost in a quantitative yield. Treatment of **4b** with MCPBA delivered the desired sulfonamide **5b**, but only in a 6% yield, while sulfinamide derivative **6b** was obtained in a yield of 77% (Scheme 1).

**Scheme 2:**
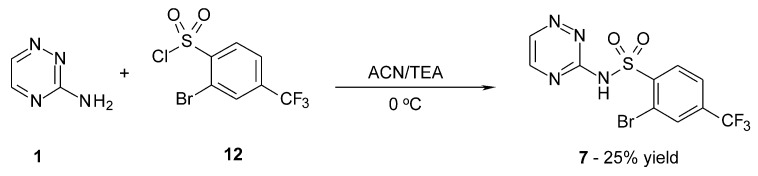
Synthesis of sulfonamide **7**.

Continuing our search for easy and efficient methods for the synthesis of 1,2,4-triazine sulfonamide derivatives, we used the more electrophilic 2-bromo-4-trifluoromethylbenzene sulfonyl chloride (**12**). Nucleophilic substitution of chloride in **12** produced sulfonamide **7** as white crystals in a 25% yield (Scheme 2). 

**Scheme 3:**
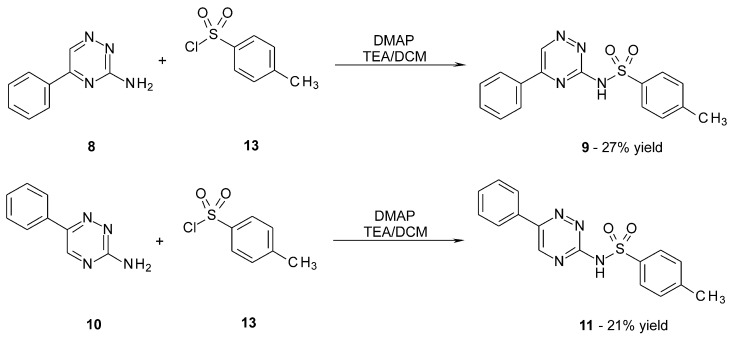
Synthesis of 1,2,4-triazine *p*-toluenesulfonamide **9** and **11**.

Due to the unsatisfactory yield of the target sulfonamide (**7**), we attempted using the less π-deficient 3-amino-5-phenyl-1,2,4-triazine (**8**) and 3-amino-6-phenyl-1,2,4-triazine (**10**) in the place of the main starting compound **1**. This resulted in the formation of new compounds **9** and **11** in a yield of 27% and 21%, respectively (Scheme 3). 

**Scheme 4:**
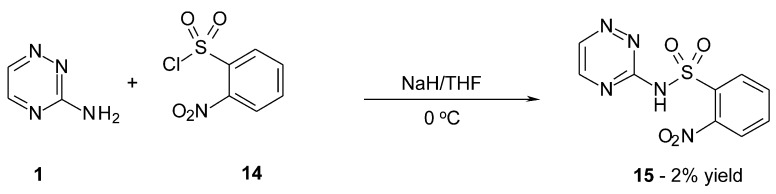
Reaction of 3-amino-1,2,4-triazine with 2-nitrobenzenesulfonyl chloride.

To expand our library of the sulfonamides investigated, 3-amino-1,2,4-triazine (**1**) was reacted with 2-nitrobenzenesulfonyl chloride (**14**) and sulfonamide (**15**) was obtained (Scheme 4). 

### 2.2. Biological Activity

**Figure 2:**
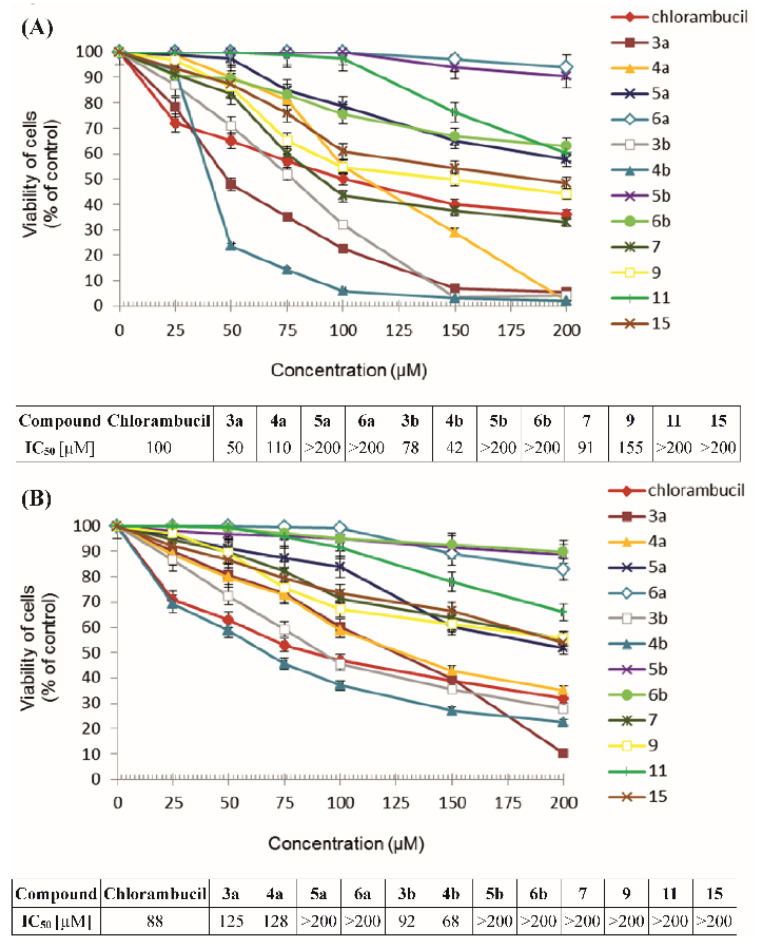
Viability of (**A**) MCF-7 and (**B**) MDA-MB-231 cells treated for 24 h with different concentrations of tested compounds (**3a**, **4a**, **5a**, **6a**, **3b**, **4b**, **5b**, **6b**, **7**, **9**, **11**, **15**) and chlorambucil. Mean ± SD values from three independent experiments (*n* = 3) done in duplicate are presented (errors were in the range of ±2).

Cell viability of breast cancer cells was determined by applying the method of Carmichael et al. using tetrazolium salt [22]. All the tested compounds were found to reduce cell viability in a dose-dependent manner, and their activity against the MCF-7 and MDA-MB-231 cells is shown in Figure 2. 

For both the cell lines, the cell growth inhibition was concertation-dependent; however, the inhibitory effect was more noticeable in the case of MCF-7 cells than MDA-MB-231 cells. The compounds that were most cytotoxic against MCF-7 cells were **4b** and **3a**, which had IC_50_ values of 42 ± 2 and 50 ± 2 μM, respectively, compared to 100 ± 2 μM calculated for chlorambucil (Figure 2A). The rest of the compounds exerted a moderate or a weak inhibitory effect on the viability of MCF-7 breast cancer cells. Among the derivatives, compound **4b** caused the highest rate of reduction in the viability of MDA-MB-231 cells, with an IC_50_ value of 68 ± 2 μM compared to that of 88 ± 2 μM determined for chlorambucil (Figure 2B). 

**Figure 3:**
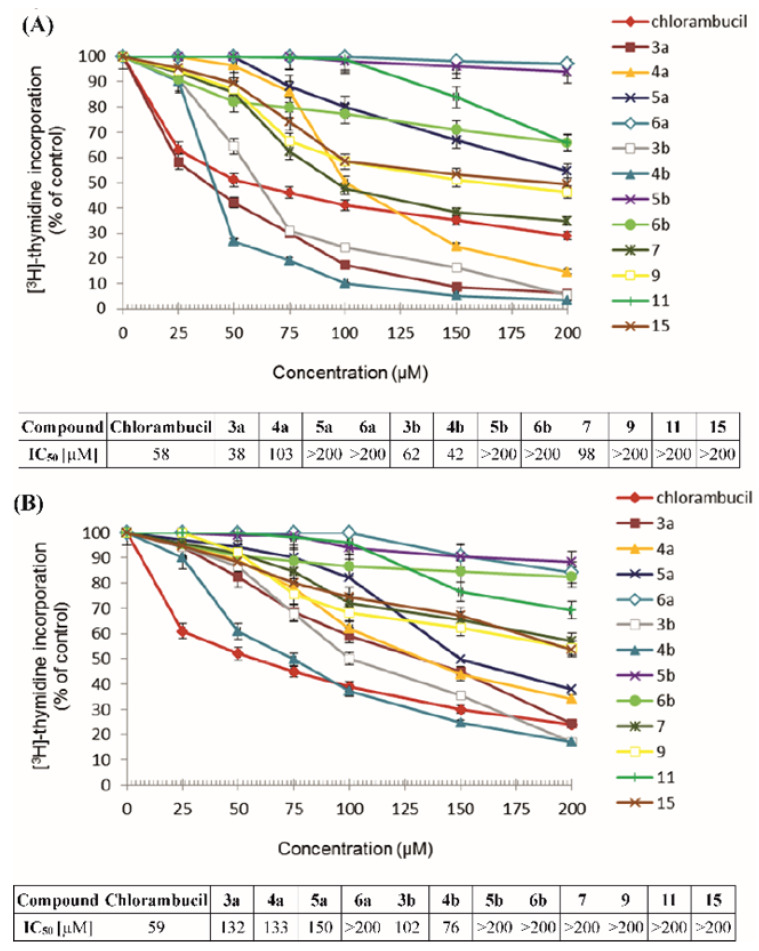
Antiproliferative effects of tested compounds (**3a**, **4a**, **5a**, **6a**, **3b**, **4b**, **5b**, **6b**, **7**, **9**, **11**, **15**) and chlorambucil in cultured (**A**) MCF-7 and (**B**) MDA-MB-231 cells as measured by inhibition of [^3^H]-thymidine incorporation into DNA. Mean ± SD values from three independent experiments (*n* = 3) done in duplicate are presented (errors were in the range of ±2).

To prove that all the tested compounds exhibited antiproliferative activity, we analyzed the DNA synthesis in cells in the presence of novel sulfonamide derivatives **3a**–**15** and chlorambucil (Figure 3). 

Most of the tested compounds showed concentration-dependent activity, but with different levels of potency. In particular, we observed that five compounds **3a**, **4a**, **3b**, **4b**, and **7** showed a higher or similar inhibitory effect on DNA biosynthesis in MCF-7 breast cancer cell lines compared to the reference drug (Figure 3). The concentration of these compounds that inhibited the incorporation of [^3^H]-thymidine into DNA by 50% (IC_50_) in MCF-7 cells was estimated to be 38 ± 2, 103 ± 2, 62 ± 2, 42 ± 2, and 98 ± 2 μM, respectively, while the IC_50_ value of chlorambucil was 58 ± 2 μM (Figure 3A). By contrast, in MDA-MB-231 cells, we observed that compared to the tested compounds, chlorambucil exhibited the highest inhibitory activity, and only compound **4b** showed a comparable antiproliferative potential. The concentration that was needed to inhibit [^3^H]-thymidine incorporation into DNA by 50% (IC_50_) in MDA-MB-231 breast cancer cell lines was found to be 132 ± 2 μM for **3a**, 133 ± 2 μM for **4a**, 102 ± 2 μM for **3b**, 76 ± 2 μM for **4b**, and 59 ± 2 μM for chlorambucil (Figure 3B). 

### 2.3. X-ray Analysis and Theoretical Calculations

**Figure 4:**
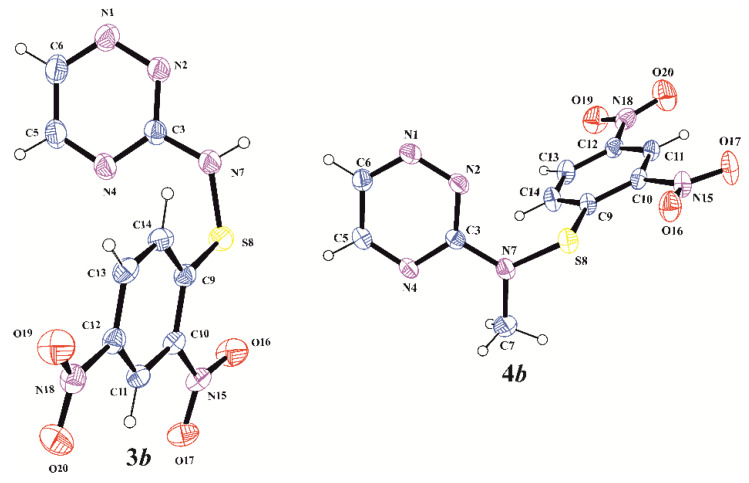
A view of the X-ray molecular structures of **3b** and **4b** with atomic labelling and displacement ellipsoids (30% probability) for non-H atoms.

The X-ray analysis of **3b** and **4b** as the model compounds confirmed their assumed molecular structures as well as the synthesis pathway of investigated sulfur derivatives of 1,2,4-triazine. The structure and conformation of the molecules **3b** and **4b** in the crystal are shown in Figure 4. 

The bond distances and angles in **3b** and **4b** are in normal ranges and are comparable to the corresponding values observed in similar structures of 2-iodo-*N*-(2-nitrophenylsulfanyl)aniline [23] and 4-iodo-*N*-(2-nitrophenylsulfanyl)aniline [24]. The N7–S8 bond length of 1.675(2) Å in **3b** and 1.699(5) Å in **4b** is slightly shorter compared to the typical N*sp*^2^–S single bond (1.710(19) Å) [25] due to its partial π character. This is confirmed by the planar configuration of the N7 atom with the sum of the valence angles around this atom of 357.52° in **3b** and 360.00° in **4b** and the C3–N7 bond length of 1.367(3) Å in **3b** and 1.387(7) Å in **4b**, intermediate between the expected single- and double-bond lengths.

**Figure 5:**
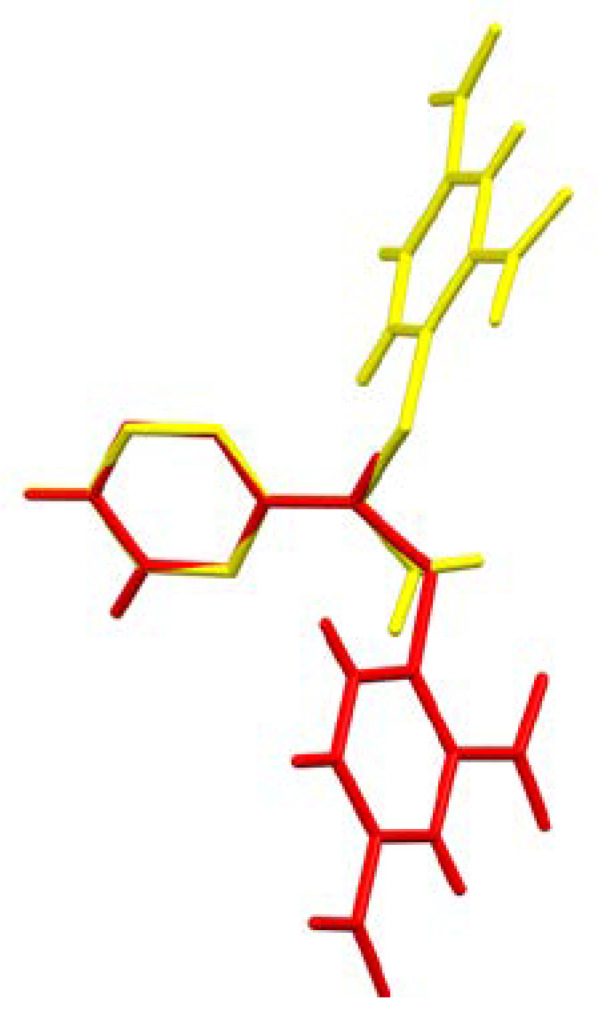
Overlay of X-ray molecules **3b** (red) and **4b** (yellow) by least-squares fitting of the atoms of 1,2,4-triazine ring (RMS = 0.0092 Å).

In both molecules, the 1,2,4-triazine and benzene rings are planar to within 0.007(2) and 0.020(2) Å in **3b** and 0.008(6) and 0.013(6) Å in **4b**, respectively, and they are inclined to each other at an angle of 71.53(6)° in **3b** and 75.09(17)° in **4b**. The torsion angles N2–C3–N7–S8 of −166.25(15)° in **3b** and 10.8(7)° in **4b**, C3–N7–S8–C9 of −81.61(19)° in **3b** and −86.2(5)^o^ in **4b** and N7–S8–C9–C10 of 169.69(16)° in **3b** and −173.6(5)° in **4b** show that the sulfonamide spacer between aromatic rings has *trans*-*gauche*-*trans* and *cis-gauche-trans* conformation in **3b** and **4b**, respectively. The torsion angles C11–C12–N15–O17 of 7.9(3)° and C11–C12–N18–O20 of −9.0(3)° in **3b** and −8.3(8) and −5.0(8)° in **4b**, respectively, show the slightly distorted coplanar positions of the nitro groups with respect to the benzene ring in these molecules. The differences in the conformations of **3b** and **4b** are presented in Figure 5 in which the overlay of both molecules is shown by fitting of the 1,2,4-triazine ring.

The characterization of the unit cell packing and intermolecular interactions [26,27] observed in the crystal structures of **3b** and **4b** is presented in the Supplementary materials. 

**Figure 6:**
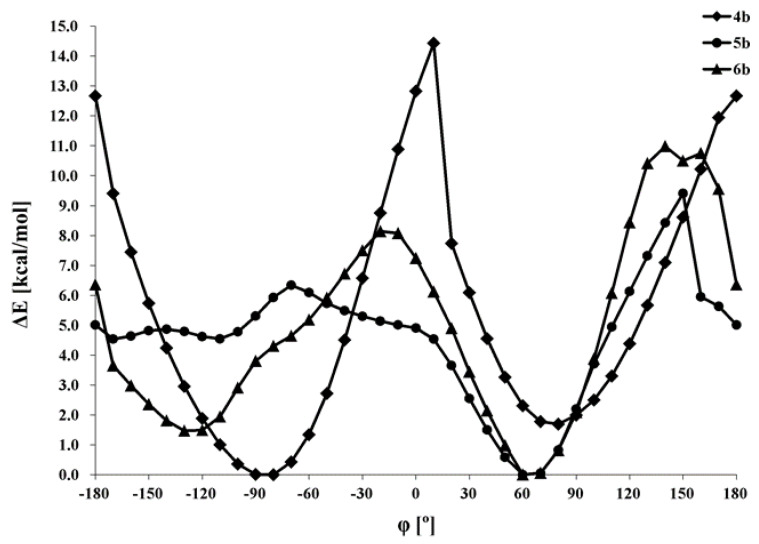
The energy effect of the free rotation on the N–S bond (C–N–S–C torsion angle) calculated for **4b**, **5b**, and **6b** using the semiempirical AM1 method.

The conformation of the molecules is an important factor associated with biological activity [28]. The conformational preferences of the investigated sulfenamides, sulfinamide, and sulfonamides were characterized by calculation of the energy effect of the free-rotation at the N–S bond for the model compounds **4b**, **5b**, and **6b** using the AM1 method. The energies of conformations were minimized, and all geometrical parameters optimized for each rotation with a 10° increment from −180 to 180° of *φ* = C–N–S–C torsion angle (Figure 6). In all analyzed cases, two minima of energy are visible for *φ* ranging from −120 to −90° and +60 to +90°, separated by the maximum energy barriers that are estimated to about 15 kcal/mol for **4b**, 10 kcal/mol for **5b**, and 11 kcal/mol for **6b**. These energy barriers indicate the practically free rotation at the N–S bond in physiological conditions. It is worth noting that the value of the torsion angle C3–N7–S8–C9 of −81.61(19) and −86.2(5)° observed in the crystal of **3b** and **4b**, respectively, is close to that (φ = −80°) theoretically calculated for minimum of energy in **4b**. The approximate perpendicular position of the bonds C–N and S–C in relation to each other results from the nearly orthogonal lone-pairs orbitals on the N and S atoms [24].

It should be noted that the results of the theoretical conformational analysis are consistent with the crystallographic data. A search of the Cambridge Structural Database (CSD; ver. 5.39, November 2017) [29,30] for the presence of the sulfonamide system N–SO_2_ connected on both sides with six-membered aromatic rings gave 930 organic structures and 1188 molecules containing this system. The distribution of the torsion angle C–N–S–C, presented as a histogram in Figure S1 (Supplementary materials) shows a good agreement with the energy diagram as a function of this angle for **6b**.

The theoretical calculations at the DFT/B3LYP/6-311+G(d,p) level performed for the molecules of all investigated compounds allowed characterization of their electron parameters, such as dipole moment, net charge distribution, energies of HOMO and LUMO frontier orbitals, and HOMO–LUMO energy gaps (Table 1). The calculations in the gaseous phase (isolated molecule) showed that the molecules are polar and have relatively large and similar values of dipole moments. Moreover, in all the molecules, the vectors of dipole moments are directed from the S–N bond toward the triazine ring. The calculated NBO net charge on the sulfur and nitrogen atoms confirms the high polarity of the S–N bond and its value increases in an absolute value as the number of oxygen atoms attached to the sulfur atom increases. 

The HOMO and LUMO frontier orbitals, as the reactivity descriptors, were calculated and are depicted in Figure S2 (Supplementary material). In **3a**, the HOMO orbital is concentrated practically on the whole molecule, while in **4b**, **5a**, and **6b**, it is distributed only on the atoms of the 1,2,4-triazine ring and partly on the S atom in the case of **4b** and **6b**. The LUMO orbital of **3a**, **4b**, **5a**, and **6b** is concentrated mainly on the benzene ring and its substituents. The energies of the HOMO and LUMO orbitals and the HOMO−LUMO energy gap are similar for all the analyzed molecules, which indicate that they have similar reactivity and stability.

The electronic parameters of molecules of all investigated compounds obtained after energy minimization and geometric parameters optimization in water solution using the CPCM model are presented in Table S1 (Supplementary materials). The NBO net charges on the atoms are very similar to those observed in the gaseous phase. However, a visible increase in the value of dipole moments of molecules is observed due to the strong polarity of the water environment. Moreover, the energy values of the frontier orbitals HOMO and LUMO, the energy gap between them, and the distribution of their wave function on atoms (Figure S3; Supplementary materials) do not differ significantly from those calculated for isolated molecules. Based on these findings, it can be concluded that the change from gaseous to water does not significantly affect the reactivity and stability of the investigated compounds.

The theoretically calculated value of logP (octanol–water partition coefficient; Table 1) is negative for compounds **3**, **4**, **5**, and **6**. This indicates the better affinity of these compounds to water than the lipid phase, in which those having two nitro groups in the benzene ring show higher hydrophilicity. The introduction of additional phenyl substituents at the 5- or 6-position of the 1,2,4-triazine ring or bromo- and trifluoromethyl groups to the benzene ring in the compounds **7**, **9**, and **11** changes their logP values from negative to positive, thereby making them highly promising drug candidates. 

### 2.4. Molecular Docking Study

In our previous study, we reported the results of molecular docking of the disulfide derivatives of 1,2,4-triazine [18]. Similarly, in the present study, we carried out this procedure for all the investigated sulfenamides (**3a**, **3b**, **4a**, and **4b)**, sulfinamides (**6a** and **6b**), and sulfonamides (**5a**, **5b**, **7**, **9**, **11**, and **15**), considering the human estrogen receptor ER*α* as the their potential molecular target. ER*α* is a major subtype of estrogen receptor (ER) found in the mammary epithelium that plays a critical role in the biology of mammary glands as well in the progression of breast cancer. ER*α* promotes the development of ER-positive breast cancer and is overexpressed in more than 70% of breast cancer cases. Moreover, several marketed selective estrogen receptor modulators, such as tamoxifen, ormeloxifene, and ratoxifene, bind to ER*α* [31,32]. It should be noted that the *N*-[(1R)-3-(4-hydroxyphenyl)-1-methylpropyl]-2-[2-phenyl-6-(2-piperidin-1-ylethoxy)-1H-indol-3-yl]-acetamide (IOG) occurring as the ligand in complex with ER*α* used in the molecular docking procedure was found to be α-selective and acted as antagonist of estradiol activity in MCF-7 cancer cells [33]. Literature reports on molecular docking of sulfonamides with antitumor activity in breast cancer to ER*α* are also known [34]. Therefore, the ER*α* was chosen for molecular docking simulation. 

The molecular docking study showed that all investigated ligands bind to the active site of ER*α*. The values of the ChemPLP scoring functions and the amino acids involved in the hydrogen bonds are presented in Table 2.

**Figure 7:**
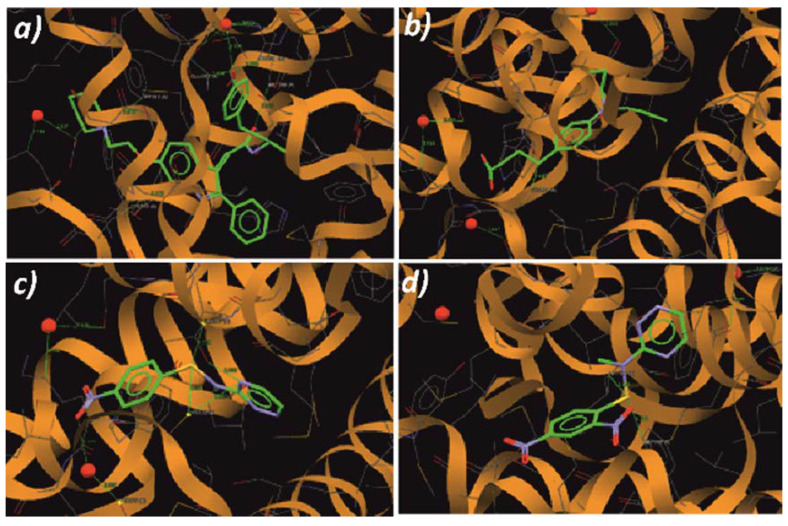
A view of the interaction between (**a**) IOG, (**b**) chlorambucil, (**c**) **4a**, and (**d**) **4b** and the binding site of ER*α*.

The redocking of IOG ligand after its removal from the binding site of ER*α* gave the best fit to the binding site with the reference value of the ChemPLP scoring function of 137.93 and showed that the O–H...O, N–H...O, S–H...C, C–H...N, and C–H...O interactions of IOG with amino acid residues in the active site are similar to those observed in the crystalline state (Figure 7a). Docking of the chlorambucil molecule as reference compound in the anticancer tests resulted in a ChemPLP scoring function of 76.79 and indicated the weak hydrogen bond S–H...C, which binds it to the active pocket (Figure 7b).

The results of docking the investigated ligands to the 2IOG protein are presented in Table 2. The values of the ChemPLP scoring function for all ligands are within a narrow range, with the highest value of 75.53 obtained for **4a** and the smallest value of 58.28 estimated for **5a**. These values are significantly smaller than that obtained for IOG, the known ERα antagonist. For the most active compound **4b**, the scoring function is 61.50. The values of the scoring function were higher for the sulfonamide derivatives **9** and **11** with an additional phenyl substituent in the 1,2,4-triazine ring and for sulfenamide derivatives **3a**, **3b**, **4a**, and **4b**.

Analysis of the intermolecular interactions between the investigated ligands and the amino acid residues in the active site of ER*α* (Table 2) shows that the nitrogen atoms of the 1,2,4-triazine ring N1 in **6a** and **6b**, and N4 in **3a**, **3b**, and **4a**, oxygen atoms of the nitro groups in **3a**, **3b**, **4a**, and **6a**, and oxygen atoms of the sulfonyl group in **9** and **11** acted as proton acceptors in the formed hydrogen bonds. Compound **4a** interacts with Tyr526 by forming hydrogen bonds through the water molecule present in the active site of ER*α*. Moreover, a lack of interactions was observed between the amino acid residues of the binding pocket and the amino nitrogen atom of the S–N linker. Only in the case of sulfenamide derivatives **3a**, **4a**, and **4b**, is the sulfur atom linked with the active site by hydrogen bonds. The amino acids that are most often implicated in hydrogen bonding interactions with the tested ligands are Cys530 (via a thiol group) and Thr347 (via a hydroxyl group). Figure 7c shows the interactions of molecule **4a**, which is best fitted to the binding site of ER*α*. This ligand is bound to the active site by the hydrogen bonds S...H–S, S...H–C, N...H–S and N...H–O with Cys530 and Thr347. Additionally, the nitro group is bound to Tyr526 through a water molecule. The most active compound **4b** in the anticancer assays is bound to the active site by means of hydrogen bonds S... H–C with Leu384 and Met388 (Figure 7d).

## 3. Materials and Methods

### 3.1. General Methods

Melting points were determined on Boethius melting point apparatus and are uncorrected. The ^1^H-NMR spectra were recorded on a Varian Gemini 400 MHz spectrometer (Bruker, Germany) with TMS as internal standard in deuterated solvents. The chemical shifts are given in δ (ppm). Mass spectra were measured on AMD 604 spectrometer (Agilent, Santa Clara, NJ, USA). Compound **1** was prepared according to a previously reported procedure [16].

#### 3.1.1. Synthesis of 3-Sulfenyl-*P*-Nitro-Phenyl-1,2,4-Triazine (**3a**)

A round-bottom flask was charged with 3-amine-1,2,4-triazine (0.37 g, 3.8 mmol), 4-nitro-benzensulfenyl chloride (0.72 g, 3.8 mmol), and THF (28 mL). The resulting reaction mixture was stirred at 0 °C for 0.5 h and then the mixture was allowed to reach room temperature and stirred for 24 h. Then, the solvent was evaporated and the residue was purified on the column with SiO_2_ using CH_2_Cl_2_:CH_3_OH (20:1) as eluent to give light yellow solid in 46% yield, m.p. 195–196 °C. ^1^H-NMR (400 MHz, CDCl_3_) δ ppm: 6.92 (s, 1H), 7.34 (dd, 2H, *J*_1_ = 2, *J*_2_ = 8.8), 8.17 (dd, 2H, *J*_1_ = 2, *J*_2_ = 8.8), 8.37 (d, 1H, *J* = 2.4), 8.91 (d, 1H, *J* = 2.4). ^13^C-NMR (100 MHz, CDCl_3_) δ ppm: 122.3, 124.3, 124.6, 144.4, 148.7, 150.0. HR-MS (ESI *m/z*) calcd. for C_4_H_8_N_5_O_2_S (M+H)^+^ 250.03932. Found 250.03944. IR (KBr) cm^−1^: 3133, 3096, 3047, 1498, 1482,1437, 1339, 852.

#### 3.1.2. Synthesis of 3-Sulfenyl-*N*-Methyl-*P*-Nitrofenyl-1,2,4-Triazine (**4a**)

A round-bottom flask was charged with NaH (0.06 g, 1.52 mmol, 60% suspension in mineral oil) and a solution of 3-sulfenyl-*p*-nitro-phenyl-1,2,4-triazine (0.40 g, 1.52 mmol) in DMF (12 mL) was added dropwise at 0 °C. After few minutes of stirring CH_3_I (0.09 mL, 1.52 mmol) was added. The resulted reaction mixture was stirred under argon at 0 °C. After 2 h, ice water was poured into the reaction mixture. The precipitate was filtered off and purified by column chromatography using CH_2_Cl_2_:CH_3_COCH_3_ (100:1) as eluent. Expected product was isolated in 49% yield as yellow solid, m.p. 120–121 °C. ^1^H-NMR (400 MHz, CDCl_3_) δ ppm: (s, 3H), 7.16 (dd, 2H, *J*_1_ = 32.4, *J*_2_ = 9.2), 8.15 (dd, 2H, *J*_1_ = 32.4, *J*_2_ = 9.2), 8.35 (d, 1H, *J* = 2.4), 8.84 (d, 1 H, *J* = 2.4). ^13^C-NMR (100 MHz, CDCl_3_) δ ppm: 42.7, 122.0, 124.4, 143.0, 145.8, 148.0, 149.5, 163.5. HR-MS (ESI *m/z*) calcd. for C_10_H_10_N_5_O_5_S (M+H)^+^ 264.05497. Found 264.05449.

#### 3.1.3. Synthesis of 3-Sulfonyl-*N*-Methyl-*P*-Nitrophenyl-1,2,4-Triazine (**5a**)

A solution of 3-sulfenyl-*N*-methyl-*p*-nitrophenyl-1,2,4-triazine (0.014 g, 0.05 mmol) in anhydrous CH_2_Cl_2_ (2 mL) was cooled to −10 °C and MCPBA (0.025 g, 0.1 mmol) was added by portions. The reaction mixture was stirred at −10 °C until the starting compounds disappeared. Then, the reaction mixture was stirred at room temperature for 24 h. The solvent was evaporated. To the residue, 20% solution of NaHCO_3_ was added until the pH reached 7. The water layer was extracted by CH_2_Cl_2_. The organic layer was dried over Na_2_SO_4_. The solvent was evaporated, and the crude products were purified by column chromatography using CH_2_Cl_2_:CH_3_COCH_3_ (100:1) as eluent. White crystals were obtained in 25% yield, m.p. 138–146 °C. ^1^H-NMR (400 MHz, CDCl_3_) δ ppm: 3.82 (s, 3H), 8.30 (dd, 2H, *J*_1_ = 2, *J*_2_ = 9.3), 8.35 (dd, 2H, *J*_1_ = 2, *J*_2_ = 9.3), 8.37 (d, 1H, *J* = 2), 8.95 (d, 1H, *J* = 2.4). ^13^C-NMR (100 MHz, CDCl_3_) δ ppm: 34.4, 124.0, 129.9, 145.2, 145,3, 147.5, 148.4, 159.3. HR-MS (ESI *m/z*) calcd. for C_10_H_10_N_5_O_4_S (M+H)^+^ 296.04480. Found 296.04448. IR (KBr) cm^−1^ 1607, 1536, 1352, 1366, 854.

#### 3.1.4. Synthesis of 4-Nitro-*N*-Methyl-1,2,4-Triazine-3-Yl-Benzenesulfinamide (**6a**)

As second product was obtained the sulfinyl derivative in 2.3% yield. M.p. 140–160 °C. ^1^H-NMR (400 MHz, CDCl_3_) δ ppm: 3.10 (s, 3H), 7.99 (d, 2H, *J* = 9.2 Hz), 8.41 (d, 2H, *J* = 8.8 Hz), 8.44 (d, 1H, *J* = 2.8 Hz), 8.98 (d, 1H, *J* = 2.4 Hz). ^13^C-NMR (100 MHz, CDCl_3_) δ ppm: 50.6, 124.3, 124.5, 126.7, 126.8, 144.5, 149.2, 149.3. HR-MS (ESI *m/z*) calcd. for C_10_H_10_N_5_S_1_O_3_ (M+H)^+^ 280.04989, found 280.04989.

#### 3.1.5. Synthesis of 3-Sulfenyl-2,4-Dinitrophenyl-1,2,4-Triazine (**3b**)

A round-bottom flask was charged with 3-amine-1,2,4-triazine (0.20 g, 2.0 mmol), 2,4-dinitro-benzenesulfenyl chloride (0.49 g, 2.0 mmol) and THF (20 mL). The resulting reaction mixture was stirred for 0.5 hr at 0 °C and then the mixture was allowed to room temperature. After 24 h the solvent was evaporated. The residue was purified by column chromatography using CH_2_Cl_2_:CH_3_COCH_3_ (100:1) as eluent to give light yellow solid in 50% yield, m.p. 224–226 °C. ^1^H-NMR (400 MHz, CDCl_3_) δ ppm: 7.14 (s, 1H, NH), 8.98 (d, 1H, *J* = 2.4), 8.52 (d, 1H, *J* = 2.4), 8.47 (s, 1H), 8.44 (dd, 2H, *J*_1_ = 2.4, *J*_2_ = 20), 8.19 (s, 1H). ^13^C-NMR (100 MHz, CDCl_3_) δ ppm: 121.4, 125.7, 128.2, 140.6, 141.8, 144.5, 144.7, 149.9, 150.8. HR-MS (ESI *m/z*) calcd. for C_9_H_7_N_6_SO_4_ (M+H)^+^ 295.02440. Found 295.02469. IR (KBr) cm^−1^ 3313, 3102, 1648, 1559, 1475, 1390, 1342, 856.

#### 3.1.6. Synthesis of 3-sulfenyl-N-methyl-2,4-dinitrophenyl-1,2,4-triazine (**4b**)

A round-bottom flask was charged with NaH (0.12 g, 4.8 mmol, 60% suspension in mineral oil) and a solution of 3-sulfenyl-*p*-2,4-dinitrophenyl-1,2,4-triazine (0.70 g, 2.38 mmol) in DMF (21 mL) was added dropwise at 0 °C. After few minutes of stirring CH_3_I (0.22 mL, 1.58 mmol) was added. The resulted reaction mixture was stirred under argon at 0 °C. After 2 h, ice water was poured into to the reaction mixture. The precipitate was filtered off and purified by column chromatography using CH_2_Cl_2_:CH_3_COCH_3_ (100:1) as eluent to give **4b** (0.69 g, 95% yield) as yellow solid, m.p. 146–147 °C. ^1^H NMR (400 MHz, CDCl_3_) δ ppm: 3.75 (s, CH_3_), 7.35 (d, 1H, *J* = 9.2), 8.32 (dd, 2H, *J*_1_ = 11.6, *J*_2_ = 2.4), 8.86 (d, 1H, *J* = 2.4), 9.13 (d, 1H, *J* = 2.4). ^13^C-NMR (100 MHz, CDCl_3_) δ ppm: 41.1, 121.7, 124.6, 127.8, 142.2, 143.3, 145.2, 149.5, 149.6, 162.8.

#### 3.1.7. Synthesis of 2,4-Dinitro-*N*-Methyl-1,2,4-Triazine-3-Yl-Benzenesulfonamide (**5b**)

To a solution of 2,4-dinitro-*N*-methyl-1,2,4-triazine-3-yl-benzenesulfenamide (0.15 g, 0.49 mmol) in anhydrous CH_2_Cl_2_ (10.7 mL), MCPBA (0.48 g, 2.80 mmol) was added by portions at 0 °C. The reaction mixture was stirred at 0 °C under argon for 24 h. The solvent was evaporated and 20% solution of NaHCO_3_ was added until pH reached 7. The water layer was extracted with CH_2_Cl_2_ and the organic layers were dried under Na_2_SO_4_. After evaporation of the solvent, the crude product was purified by column chromatography using CH_2_Cl_2_:MeOH (100:1) as eluent. The expected product was obtained in 6% yield, m.p. 88–90 °C. ^1^H-NMR (400 MHz, CDCl_3_) δ w ppm: 4.00 (s, 3H), 8.11 (d, 1H, *J* = 4 Hz), 8.62 (d, 1H, *J* = 4 Hz), 8.92 (s, 1H), 8.94 (d, 1H, *J* = 8.8 Hz), 9.17 (d, 1H, *J* = 8.8 Hz). ^13^C-NMR (100 MHz, CDCl_3_) δ ppm: 34.4, 119.8, 125.7, 125.9, 127.1, 129.7, 137.2, 138.32 157.8, 160.3. HR-MS (ESI *m/z*) calcd. for C_10_H_9_N_6_S_1_O_6_ (M+H)^+^ 280.02988, found 341.02965. IR (KBr) cm^-1^ 3057, 3107, 2953-2852, 1530, 1410, 1425, 1348, 1298, 1115, 835, 731, 770, 787.

#### 3.1.8. Synthesis of 3-Sulfinyl-*N*-Methyl-2,4-Dinitrophenyl-1,2,4-Triazine (**6b**)

To a solution of 3-sulfenyl-*N*-methyl-2,4-dinitrophenyl-1,2,4-triazine (0.10 g, 0.32 mmol) in anhydrous CH_2_Cl_2_ (7 mL), MCPBA (0.20 g, 0.8 mmol) was added by portions at −10 °C. The reaction mixture was stirred at −10 °C under argon for 4 h. After the time the solvent was evaporated and 20% solution of NaHCO_3_ was added. The water layer was extracted with CH_2_Cl_2_. The crude product was purified by column chromatography using CH_2_Cl_2_:CH_3_COCH_3_ (100:1) as eluent. The product was obtained as yellow solid in 77% yield. m.p, 96–98 °C. ^1^H-NMR (400 MHz, CDCl_3_) δ ppm: 3.61 (s, 3H), 8.56 (d, 1H, *J* = 2.4), 9.19 (d, 1H, *J* = 2.4), 7.96 (dd, 2H, *J*_1_ = 2.4, *J*_2_ = 8), 7.53 (s, 1H). ^13^C-NMR (100 MHz, CDCl_3_) δ ppm: 30.9, 128.2, 129.8, 130.9, 131.1, 133.1, 134.7, 147.5, 153.8, 169.7. HR-MS (ESI *m/z*) calcd. for C_10_H_9_N_6_O_5_S (M+H)^+^ 325.03496. Found 325.03477.

#### 3.1.9. Synthesis of 2-Bromo-4-Trifluoromethyl-*N*-(1,2,4-Triazin-3-Yl)-Benzenesulfonamide (**7**)

To a solution of 3-amino-1,2,4-triazine (**1**) (0.05 g, 0.52 mmol) in acetonitryle (2 mL) triethylamine (0.05 mL, 0.57 mmol) was added. The reaction mixture was stirred at 0 °C until 2-bromo-4-trifluoromethylsulfonyl chloride (0.170 g, 0.52 mmol) in acetonitrile (1 mL) was added. Stirring was continued for further 48 h. The solvent was evaporated and the crude product was purified on column chromatography using dichloromethane:aceton (10:1) as eluent. Product was obtained in 25% yield as white crystals; m.p. 148 °C; ^1^H-NMR (CDCl_3_, 400 MHz) δ: 7.74 (s, 1H), 7.19–7.27 (m, 2H), 8.37 (s, 1H), 8.91 (s, 1H); ^13^C-NMR (100 MHz, CDCl_3_) δ: 128.5, 137.9, 138.8, 139.2, 145.2, 145.3, 147.5, 154.4, 159.7, 168.5; IR (KBr, cm^−1^) δ: 725, 854, 1378, 1330, 1536, 1607, 3250; HRMS (ESI *m/z*) calcd. for C_10_H_7_N_4_SO_2_F_3_ [M+H]^+^ 384.93992, found 384.95817.

#### 3.1.10. Synthesis of 4-Methyl-*N*-(6-Phenyl-1,2,4-Triazin-3-Yl)-Benzenesulfonamide (**9**)

A round-bottom flask was charged with 6-fenyl-3-amino-1,2,4-triazine (0.12 g, 0.75 mmol), 4-toluenesulfonyl chloride (0.14 g, 0.75 mmol) and 4-dimethyloaminopyridine (30 mol%). To the resulting mixture, triethylamine (0.05 mL) and anhydrous CH_2_Cl_2_ (6 mL) were funneled. The reaction mixture was stirred under argon at room temperature for three days. After evaporation of the solvent the crude product was purified by column chromatography on silica gel using dichloromethane: acetone (10:1) as eluent. Pure compound was obtained as white crystals in 27% yield; m.p. 93 °C; ^1^H-NMR (CDCl_3_, 400 MHz) δ: 2.45 (s, 3H), 7.37–7.45 (m, 5H), 7.83 (d, *J* = 8.8 Hz, 2H), 8.03 (d, *J* = 8.4 Hz, 2H), 8.31 (s, 1H); ^13^C-NMR (100 MHz, CDCl_3_) δ: 21.8, 118.8, 126.0, 128.7, 128.8, 128.9, 129.0, 130.4, 133.1, 147.3; IR (KBr, cm^−1^) δ: 414, 586, 1300, 1375, 1442, 1485, 1599, 3084, 3105; HRMS (ESI *m/z*) calcd. for C_16_H_14_N_4_SO_2_ [M+H]^+^ 327.09102 found 327.09380.

#### 3.1.11. Synthesis of 4-Methyl-*N*-(5-Phenyl-1,2,4-Triazin-3-Yl)-Benzenesulfonamide (**11**)

A round-bottom flask was charged with 6-fenyl-3-amino-1,2,4-triazine (0.12 g, 0.75 mmol), 4-toluenesulfonyl chloride (0.14 g, 0.75 mmol), and 4-dimethyloaminopyridine (30 mol%). To the resulting mixture, triethylamine (0.05 mL) and anhydrous CH_2_Cl_2_ (6 mL) were funneled. The reaction mixture was stirred under argon at room temperature for three days. The solvent was evaporated, and the crude product was purified by column chromatography on silica gel using dichloromethane: acetone (10:1) as eluent. Pure compound was obtained as white crystals in 21% yield; m.p. 97 °C; ^1^H-NMR (CDCl_3_, 400 MHz) δ: 2.42 (s, 3H), 7.35 (d, *J* = 8.4, 2H), 7.42–7.44 (m, 3H), 7.81–7.84 (m, 2H), 8.01 (d, *J* = 8.8 Hz, 2H), 8.07 (s, 1H); ^13^C-NMR (100 MHz, CDCl_3_) δ: 21.8, 126.7, 128.2, 128.7, 129.0, 129.9, 130.1, 133.0 135.7, 146.6, 167.5; IR (KBr, cm^−1^) δ: 420, 590, 1300, 1375, 1445, 1485, 1599, 3084, 3115; HRMS (ESI *m/z*) calcd. for C_16_H_14_N_4_SO_2_ [M+H]^+^ 327.09102 found 327.09380.

#### 3.1.12. Synthesis of 2-Nitro-*N*-1,2,4-Triazine-3-Yl-Benzenesulfonamide (**15**)

The round-bottom flask was charged with NaH (0.93 g, 3.88 mmol, 60% suspension in mineral oil) and 3-amine-1,2,4-triazine (0.20 g, 2.08 mmol) solution in THF (13 mL) was added dropwise at 0 °C. After few minutes of stirring, 2-nitrobenzenesulfonyl chloride (0.46 g, 2.18 mmol) was added. The reaction mixture was stirred at 0 °C under argon for 72 h. The solvent was evaporated and the crude product was purified by column chromatography using CH_2_Cl_2_:MeOH (10:1) as eluent. The expected product was obtained in 2% yield. M.p. 86–93 °C. ^1^H-NMR (400 MHz, CDCl_3_) δ ppm: 7.16 (dt, 1H, *J*_1_ = 1.2 Hz, *J*_2_ = 7.2 Hz), 7.70 (dt, 1H, *J*_1_ = 1.2 Hz, *J*_2_ = 7 Hz), 8.30 (dd, 1H, *J*_1_ = 1.4 Hz, *J*_2_ = 8.6 Hz), 8.99 (dd, 1H, *J*_1_ = 1.2 Hz, *J*_2_ = 8.4 Hz), 8.91 (d, 1H, *J* = 2.4 Hz), 8.42 (d, 1H, *J* = 2.8 Hz). HR-MS (ESI *m/z*) calcd. for C_9_H_8_N_5_S_1_O_4_ (M+H)^+^ 282.02915, found 282.27876. 

### 3.2. Pharmacology

#### 3.2.1. Cell Culture

Cultured normal human skin fibroblasts and MCF-7 human breast cancer cells were maintained in DMEM supplemented with 10% fetal bovine serum (FBS), 50 U/mL penicillin, 50 μg/mL streptomycin at 37 °C. Cells were cultured in Costar flasks, and subconfluent cells were detached with 0.05% trypsin and 0.02% EDTA in calcium-free phosphate buffered saline, counted in hemocytometers and plated at 5 × 10^5^ cells per well of six-well plates (Nunc) in 2 mL of growth medium (DMEM without phenol red with 10% CPSR1). Cells reached about 80% of confluency at day 3, and in most cases, such cells were used for the assays.

#### 3.2.2. Cell Viability Assay

The assay was performed according to the method of Carmichael [22] using 3-(4,5-dimethylthiazole-2-yl)-2,5-diphenyltetrazolium bromide (MTT). Confluent cells, cultured for 24 h with various concentrations of studied compounds in six-well plates were washed three times with PBS and then incubated for 4 h in 1 mL of MTT solution (0.5 mg/mL of PBS) at 37 °C in 5% CO_2_ in an incubator. The medium was removed and 1 mL of 0.1 mol/L HCl in absolute isopropanol was added to the cells attached. Absorbance of converted dye in the living cells was measured at a wavelength of 570 nm. Cell viability of breast cancer cells cultured in the presence of ligands was calculated as a percentage of controlled cells. 

#### 3.2.3. DNA Synthesis Assay

MCF-7 cells were seeded in six-well plates and were incubated with varying concentrations of **1–18** or chlorambucil and 0.5 μCi of [^3^H]-thymidine for 24 h at 37 °C [21]. The cells were then harvested by trypsinization, washed with cold phosphate-buffered saline, and centrifuged for 10 min at 1500× g several times (4–5) until the dpm in the washes were similar to the reagent control. Radioactivity was determined by liquid scintillation counting. [^3^H]-thymidine uptake was expressed as dpm/well.

### 3.3. X-ray Structure Determinations

X-ray data of **3b** were collected on the Bruker SMART APEX II CCD diffractometer; crystal sizes 0.39 × 0.06 × 0.003 mm, CuK*α* (*λ* = 1.54178 Å) radiation, *ω* scans, *T* = 296(2) K, absorption correction: multi-scan SADABS [35], *T*_min_/*T*_max_ = 0.6056/0.7529. X-ray data of **4b** were collected on the KUMA Diffraction KM-4 CCD diffractometer; crystal sizes 0.60 × 0.50 × 0.40 mm, MoK*α* (*λ* = 0.71073 Å) radiation, *ω* scans, *T* = 296(2) K, absorption correction: multiscan CrysAlisPro [36], *T*_min_/*T*_max_ of 0.8245/1.0000. The structures **3b** and **4b** were solved by for direct methods using SHELXS-2013/1 [37] and refined by full matrix least squares with SHELXL-2014/7 [37]. The N-bound H atom in **3b** was located by difference Fourier synthesis and refined freely. The remaining H atoms were positioned geometrically and treated as riding on their parent C atoms with C–H distances of 0.93 Å (aromatic). All H atoms were refined with isotropic displacement parameters taken as *U*_iso_(H) = 1.5*U*_eq_(C,N). The compound **4b** crystallizes in noncentrosymmetric space group Pna21. The absolute structure of crystal was confirmed by Flack parameter of 0.04(8) with 439 Friedel pairs [38]. All calculations were performed using WINGX version 2014.1 package [39]. CCDC-1939569 and CCDC-1939570 contain the supplementary crystallographic data for this paper. These data can be obtained free of charge at www.ccdc.cam.ac.uk/conts/retrieving.html (or from the Cambridge Crystallographic Data Centre (CCDC), 12 Union Road, Cambridge CB2 1EZ, UK; fax: +44(0) 1223 336 033; email: deposit@ccdc.cam.ac.uk). 

Crystal data of **3b**: C_9_H_6_N_6_O_4_S, M_r_ = 294.26 gmol^−1^, monoclinic, space group C2/c, a = 27.8714(6), b = 4.6957(1), c = 17.8150(2) Å, *β* = 95.211(2)°, V = 2321.91(8) Å^3^, Z = 8, D_calc_ = 1.684 g cm^−3^, F(000) = 1200, *μ*(Cu Kα) = 2.769 mm^−1^, T = 296(2)K, 6363 measured reflections (*θ* range 3.18–65.63^o^), 1981 unique reflections (R_int_ = 0.027) final R = 0.036, wR = 0.099, S = 1.023 for 1682 reflections with I > 2*σ*(I), Δ*ρ*_max_ = + 0.235 and Δ*ρ*_min_ = −0.170 eÅ^−3^.

Crystal data of **4b**: C_10_H_8_N_6_O_4_S, M_r_ = 308.28 gmol^−1^, orthorhombic, space group Pna2_1_, a = 7.3650(13), b = 8.485(2), c = 20.887(4) Å, V = 1304.6(5) Å^3^, Z = 4, D_calc_ = 1.570 g cm^−3^, F(000) = 632, *μ*(Mo K*α*) = 0.276 mm^−1^, T = 293(2)K, 3501 measured reflections (*θ* range 1.95–29.31°), 2158 unique reflections (R_int_ = 0.025) final R = 0.057, wR = 0.136, S = 1.280 for 1810 reflections with I > 2*σ*(I), Δ*ρ*_max_ = +0.434 and Δ*ρ*_min_ = −0.394 eÅ^−3^.

### 3.4. Theoretical Calculation

The theoretical calculations were performed for all investigated compounds at the DFT/B3LYP/6-311++G(d,p) level using the Gaussian 03 program [40]. The structures were fully optimized without any symmetry constraints and the initial geometries were built from the crystallographic data of **3b** and **4b**. The calculations were carried out in gaseous phase and water solution (The Conductor Polarizable Continuum Model; CPCM [41]). GaussView 4.1 program was used to construct and visualization of the HOMO and LUMO orbitals [42]. The conformational preferences of **4b**, **5b**, and **6b** were calculated using semiempirical AM1 method implemented in GAUSSIAN 03.

### 3.5. Molecular Docking

The crystal structure of the human estrogen receptor alpha (ER*α*) in complex with *N*-[(1*R*)-3-(4-hydroxyphenyl)-1-methylpropyl]-2-[2-phenyl-6-(2-piperidin-1-ylethoxy)-1*H*-indol-3-yl]acetamide (IOG) was downloaded from Protein Data Bank (PDB ID: 2IOG; resolution 1.6 Å) [34]. The molecular docking studies were performed using the GOLD Suite v.5.6.3 [43]. Preparation of the receptor comprising addition of hydrogens and extraction of original ligand from the protein active site was done with GOLD as per default settings. The crystal waters were also deleted with the exception of HOH605, HOH645, HOH657, and HOH734, which occur in the active site of ER*α*. Moreover, the molecules HOH605 and HOH645 are involved in the interaction of the ligand IOG with amino acids residues of the receptor. The binding site was determined using the previous knowledge of the original ligand interaction site [34]. The reference ligand (IOG) was removed from X-ray structure of its protein–ligand complex (2IOG) and docked back into its binding site with the RMSD values of 0.662 Å for ChemPLP, confirming that prediction of the binding mode was successful. The docking procedure was carried out with flexible ligands and rigid the amino acid residues of the binding site of receptor. The water molecules could spin and translate to optimize the orientation of the hydrogen atoms as well as the water molecule’s position. For the simulation runs, default parameter values were used. The selection of atoms in the active site within 6 Å of original ligands was chosen as default. The ChemPLP was selected as the scoring function to rank the compounds to be investigated. Solutions and protein–ligand interactions were analyzed using Hermes v1.8.2 [43]. 

## 4. Conclusions

In this study, new sulfonamide derivatives of 1,2,4-triazine were synthesized via a direct method using chlorosulfonyl chloride and via a two-step process after obtaining a sulfenamide intermediate. The last step involved a reaction between chlorosulfenyl and amino-1,2,4-triazine. This product was alkylated and then oxidized to sulfonamide. 

The sulfur derivatives of 1,2,4-triazine were further tested in vitro for their anticancer activity against the human breast cancer cell lines MCF-7 and MDA-MB-231. Biological studies showed that the cytotoxic activity of **4b**, **3a**, **3b**, and **7** against MCF-7 cell line was higher than that of reference compound chlorambucil. Similarly, higher antiproliferative activity was observed for compounds **3a**, **4a**, **3b**, **4b**, and **7** in comparison with chlorambucil. In the case of the cell line MDA-MB-231, only the cytotoxic and antiproliferative activities of compound **4b** were comparable to those of chlorambucil. 

X-ray analysis confirmed the assumed molecular structure and the synthesis pathway of investigated sulfur derivatives of 1,2,4-triazine and provided structural data for molecular modeling. Conformational analysis showed the high degree of freedom of rotation of the S–N bond and indicated the preferential conformation of the S–N spacer with the perpendicular position of the N–C and C–S bonds with respect to each other.

Theoretical calculation at the DFT/B3LYP/6-311++G(d,p) level showed that all the investigated compounds have similar reactivity and stability. The differences in the electronic structure of molecules are mainly related to the degree of oxidation of the sulphur atom. The theoretically calculated logP values indicated **7**, **9**, and **11** as highly promising drug candidates.

The results of docking of the investigated compounds to ER*α* revealed that compound **4a**, which showed weak antiproliferative activity against the MCF-7 cell line, had the best fit to the binding site of the protein. Moreover, sulfonamides **9** and **11**, with an additional phenyl substituent in the 1,2,4-triazine, did not exhibited antiproliferative activity against the MCF-7 cells, but they showed similar values of the ChemPLP scoring function to the reference chlorambucil, and higher values than the other investigated compounds. Among the ligand molecules, the sulfur atom of the S–N spacer and the nitrogen atoms of the 1,2,4- triazine ring play a crucial role in the interactions with the active site of ER*α*. The amino acids Cys530 and Thr347 are bound to the molecules of the tested ligands in most of the cases. 

A comparison of the values of the ChemPLP scoring function obtained for IOG, chlrambucil and all investigated sulphur derivatives of 1,2,4-triazine may reveal that the mechanism behind the anticancer activity of the tested compounds is different from the mechanism of action of the IOG ligand.

## Figures and Tables

**Table 1:** The dipole moments, *D_m_* (D), NBO atomic charges, (e), energies of HOMO and LUMO orbitals, *E*_HOMO_ and *E*_LUMO_ (eV) calculated at DFT/B3LYP/6-311++G(d,p) level in the gaseous phase and calculated logP coefficient for **3ab**–**6ab**, **7**, **9**, **11**, and **15**.

Compound	*D_m_*	NBO Charge		*E*_HOMO_	*E*_LUMO_	Δ*E*	log*P*
		S	N				
**3a**	4.697	+0.523	−0.767	−7.04	−2.90	4.14	−0.97
**3b**	4.198	+0.613	−0.789	−7.27	−3.55	3.72	−4.88
**4a**	4.564	+0.532	−0.636	−6.84	−2.87	3.97	−1.20
**4b**	4.145	+0.630	−0.658	−7.07	−3.51	3.56	−5.11
**5a**	2.431	+2.217	−0.701	−7.17	−3.46	3.71	−2.15
**5b**	3.778	+2.230	−0.705	−7.32	−3.87	3.45	−6.06
**6a**	3.618	+1.408	−0.657	−7.02	−3.30	3.72	−1.87
**6b**	1.275	+1.444	−0.655	−7.14	−3.81	3.33	−5.78
**7**	4.069	+2.208	−0.854	−7.37	−2.89	4.48	3.67
**9**	7.042	+2.222	−0.830	−6.86	−2.60	4.26	4.08
**11**	6.417	+2.211	−0.835	−6.79	−2.47	4.32	4.08
**15**	7.090	+2.218	−0.841	−7.22	−2.73	4.49	−1.91

**Table 2:** The results of the molecular docking of ligands **3ab**–**6ab**, **7**, **9**, **11**, and **15** to estrogen receptor alpha (ER*α*).

Compound	ChemPLP	Ligand–Amino Acids Interactions
**3a**	63.03	N4...H–S(Cys530), S8...H–C(Ala350), (N=)O...H–C(Leu536)
**3b**	62.07	(N=)O...H–C(His524), (N=)O...H–O(Thr347), S8...H–S(Cys530) S8...H–C(Thr347), N4...H–S(Cys530)
**4a**	75.53	N4...H–O(Thr347), (N=)O...H–O-H...H–O(Tyr526)
**4b**	61.50	S8...H–C(Leu384), S8...H–C(Met388)
**5a**	58.52	-
**5b**	58.28	-
**6a**	60.40	N1...H–S(Cys530), N1...H–O(Thr347), (N=)O...H–N(Arg394)
**6b**	59.62	N1...H–O(Thr347)
**7**	65.21	-
**9**	75.26	(S8=)O...H–C(Leu384)
**11**	72.61	(S8=)O...H–C(Ala350), (Ph)C–H…O(Glu419)
**15**	59.92	-
**IOG**	137.93	O…H–O(Glu353), O…H–N(Arg394), C…H–S(Cys530), N…H–C(Trp383), O…H–C(Met388)
**Chlorambucil**	76.79	C…H–S(Cys530)

## Supplementary Materials

The following are available online, Figure S1: Histogram of torsion angle C–N–S–C in sulfonamide system, Figure S2: Schematic drawings of the HOMO and LUMO orbitals of **3a**, **4b**, **5a** and **6b** as calculated in the gaseous phase using DFT/B3LYP/6-311++G(d,p) method, Figure S3: Schematic drawings of the HOMO and LUMO orbitals of **3a**, **4b**, **5a** and **6b** as calculated in the water solution using DFT/B3LYP/6-311++G(d,p) method, Table S1: The dipole moments, *D_m_* (D), NBO atomic charges, (*e*), energies of HOMO and LUMO orbitals, *E_HOMO_* and *E_LUMO_* (eV) calculated at DFT/B3LYP/6-311++G(d,p) level in the water solution for **3ab**–**6ab**, **7**, **9**, **11** and **15**.
