# Supplementary material for: 1,2,4-Triazine Sulfonamides: Synthesis by Sulfenamide Intermediates, In Vitro Anticancer Screening, Structural Characterization, and Molecular Docking Study
Source: Molecules. 2020 May 16;25(10):2324. doi: 10.3390/molecules25102324 (PMC7288137; doi:10.3390/molecules25102324)
Supplement: Supplementary file 1 [file molecules-25-02324-s001.pdf]

Supplementary material for publication online

Article

1,2,4-Triazine Sulfonamides: Synthesis by Sulfenamide Intermediates, in Vitro Anticancer Screening, Structural Characterization and Molecular Docking Study

Danuta Branowska [1], Zbigniew Karczmarzyk [1,*], Ewa Wolińska [1], Waldemar Wysocki [1], Maja Morawiak [2], Zofia Urbańczyk-Lipkowska [2], Anna Bielawska [3] and Krzysztof Bielawski [3]

[1] Siedlce University of Natural Sciences and Humanities, Faculty of Exact and Natural Sciences, 3 Maja 54, 08-110 Siedlce, Poland; danuta.branowska@uph.edu.pl (D.B.); ewa.wolinska@uph.edu.pl (E.W.); waldemar.wysocki@uph.edu.pl (W.W.)
[2] Polish Academy of Sciences, Institute of Organic Chemistry, Kasprzaka 44/52, 01-224 Warsaw, Poland; maja.morawiak@icho.edu.pl (M.M.); zofia.lipkowska@icho.edu.pl (Z.U.-L.)
[3] Medical University of Bialystok, Department of Medicinal Chemistry and Drug Technology, J. Kilinskiego 1, 15-089 Bialystok, Poland; aniabiel@umb.edu.pl (A.B.); kbiel@umb.edu.pl (K.B.)
* Correspondence: zbigniew.karczmarzyk@uph.edu.pl; Tel.: +48-25-643-1017

Description of the crystal structures 3b and 4b

In the crystal structure of **3b** the inversion related molecules form molecular dimers through pair of intermolecular N7–H7…N2^{i} hydrogen bonds [N7–H7 = 0.84(3), H7…N2 = 2.19(3), N7…N2 = 2.935(3) Å, N7–H7…N2 = 172(3)° and *i* = 1/2-x, -1/2-y, 1-z].

Due to the lack of classical proton-donor groups ,the molecular packing in the crystal of **4b** is influenced by the presence of the weak C–H…X (X = O, N) hydrogen bonds: C6–H6…O20^{i} [C6–H6 = 0.93, H6…O20 = 2.56, C6…O20 = 3.190(9) Å, C6–H6…O20 = 126° and *i* = 3/2-x, 1/2+y, -1/2+z], C14–H14…N1^{ii} [C14–H14 = 0.93, H14…N1 = 2.53, C14…N1 = 3.361(9) Å, C14–H14…N1 = 149° and *ii* = 1/2+x, 3/2-y, z] and C14–H14…N2^{ii} [C14–H14 = 0.93, H14…N2 = 2.59, C14…N2 = 3.316(8) Å, C14–H14…N2 = 135°].

A search of the Cambridge Structural Database (CSD; ver. 5.39, November 2017)

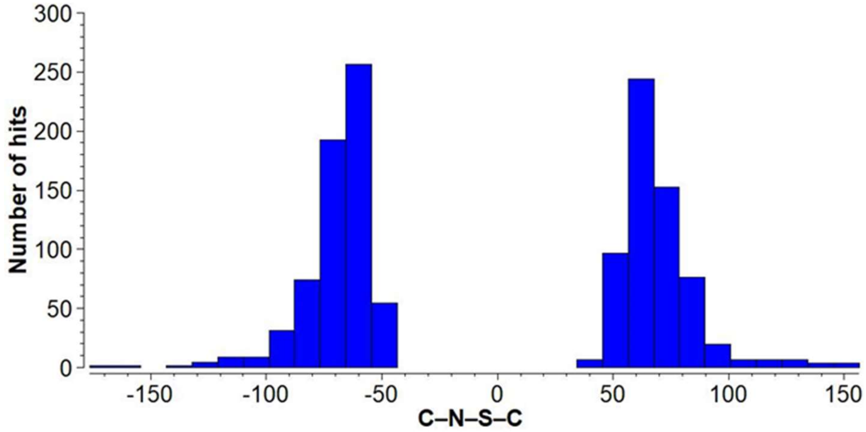

Figure S1. Histogram of torsion angle C–N–S–C in sulfonamide system.

Results of theoretical calculation on DFT/B3LYP/6-311G++(d,p) level for molecules 3ab–6ab, 7, 9, 11 and 15 in water solution using CPCM model.

Table S1. The dipole moments, D_m (D), NBO atomic charges, (*e*), energies of HOMO and LUMO orbitals, E_{HOMO} and E_{LUMO} (eV) calculated at DFT/B3LYP/6-311++G(d,p) level in the water solution for **3ab–6ab**, **7**, **9**, **11** and **15**.

Compound	D_m	NBO charge		E_{HOMO}	E_{LUMO}	ΔE
		S	N			
**3a**	6.404	+0.532	−0.772	−6.76	−3.07	3.69
**3b**	5.587	+0.615	−0.790	−7.04	−3.28	3.66
**4a**	6.089	+0.542	−0.633	−6.68	−3.08	3.60
**4b**	5.357	+0.627	−0.651	−6.91	−3.40	3.51
**5a**	3.501	+2.228	−0.699	−7.09	−3.42	3.69
**5b**	6.046	+2.240	−0.704	−7.19	−3.61	3.58
**6a**	5.698	+1.422	−0.668	−6.95	−3.31	3.64
**6b**	1.062	+1.453	−0.650	−7.00	−3.61	3.39
**7**	6.310	+2.229	−0.864	−7.25	−2.61	4.64
**9**	7.042	+2.222	−0.830	−6.86	−2.60	4.26
**11**	9.379	+2.213	−0.850	−6.77	−2.50	4.27
**15**	12.064	+2.233	−0.860	−7.23	−3.22	4.01

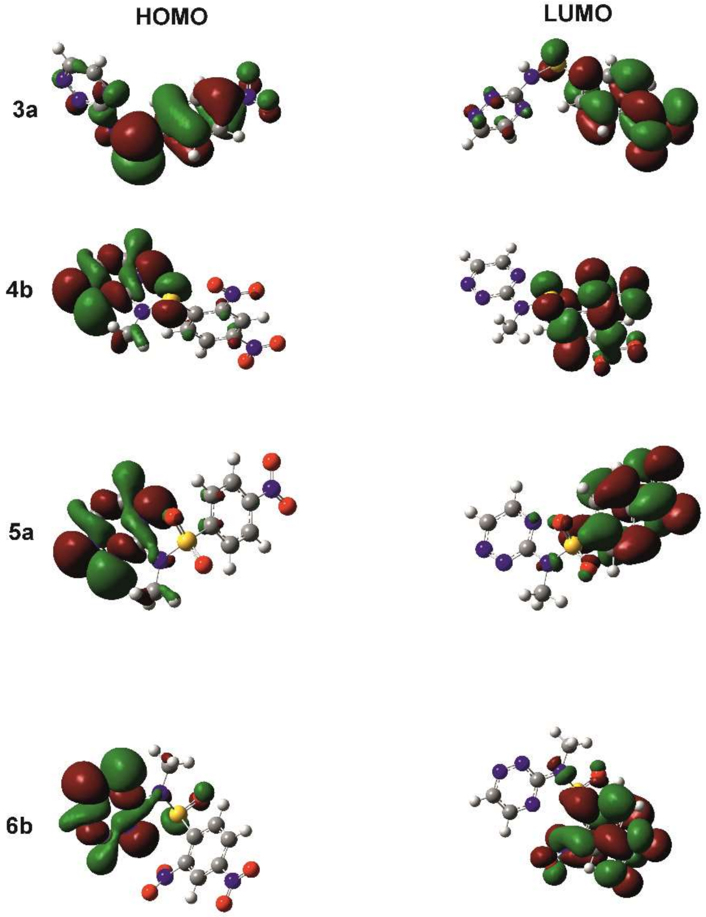

Figure S2. Schematic drawings of the HOMO and LUMO orbitals of **3a**, **4b**, **5a** and **6b** as calculated in the gaseous phase using DFT/B3LYP/6-311++G(d,p) method.

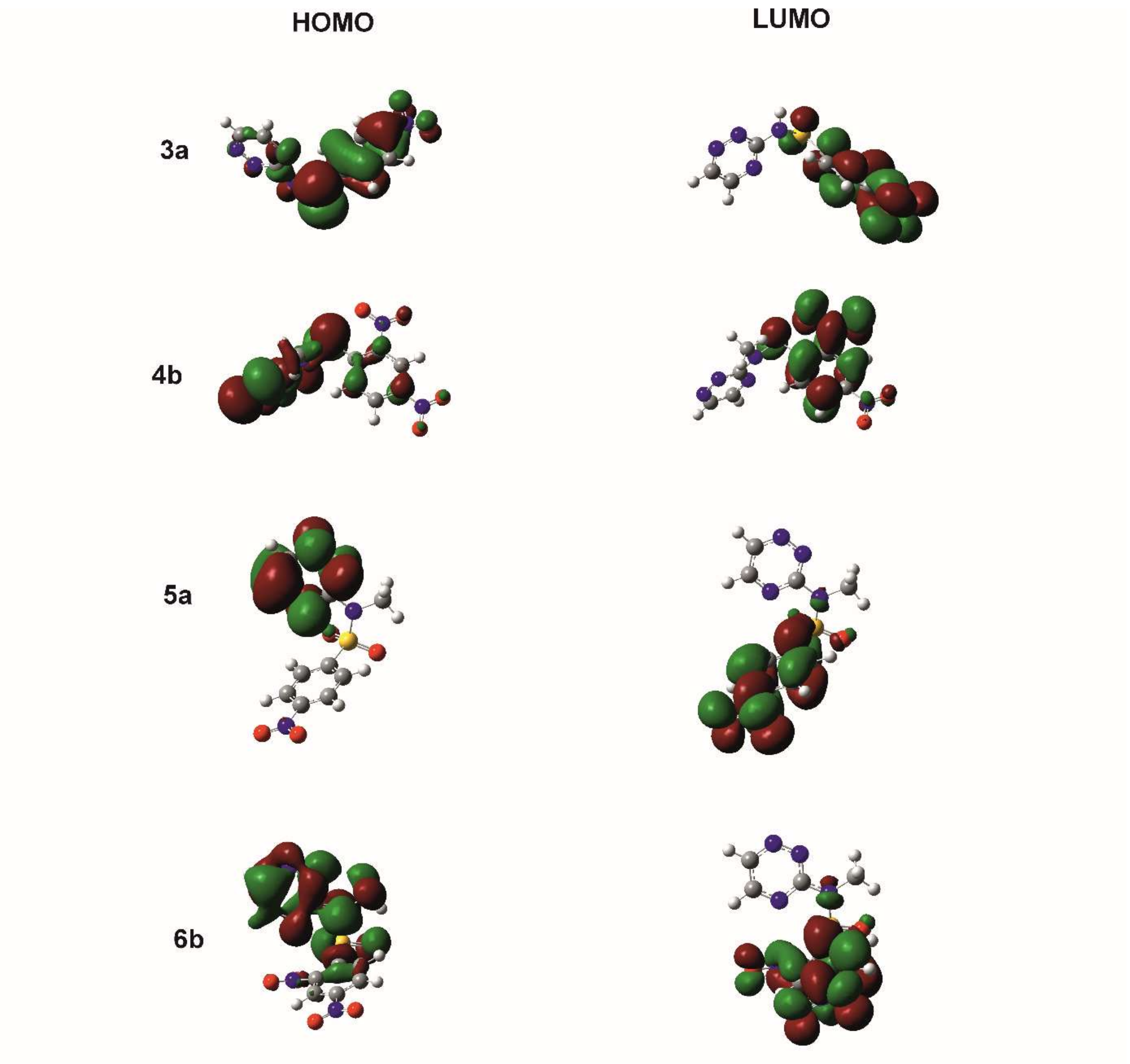

Figure S3. Schematic drawings of the HOMO and LUMO orbitals of **3a**, **4b**, **5a** and **6b** as calculated in the water solution using DFT/B3LYP/6-311++G(d,p) method.
